# Organ donation: the reality of an intensive care unit in Portugal

**DOI:** 10.5935/0103-507X.20180040

**Published:** 2018

**Authors:** Carla Sofia Lopes da Eira, Maria Inês Trindade de Barros, Ana Maria Pina de Albuquerque

**Affiliations:** 1 Serviço de Medicina, Centro Hospitalar Tondela-Viseu - Viseu, Portugal.; 2 Unidade de Cuidados Intensivos Polivalente, Centro Hospitalar Tondela-Viseu - Viseu, Portugal.

**Keywords:** Tissue donors, Brain death, Intensive care units

## Abstract

**Objective:**

To clinically and demographically characterize potential organ donors admitted to a general intensive care unit and analyze data on donated organs.

**Methods:**

This retrospective study was conducted from 2010 to 2015 and analyzed demographic and clinical variables and the number of harvested organs and tissues.

**Results:**

A total of 92 potential organ donors were identified, of whom eight were non-effective donors and 84 were effective donors (59.5% were expanded criteria donors). The mean age of the potential donors was 60.7 years, and the majority were men. Hemorrhagic stroke accounted for 55.4% of brain deaths. The most common blood type among the donors was A Rh+ (43.5%), and the most common comorbidity was arterial hypertension (43.3%). The most frequently collected organs were the kidneys (84.5%) and liver (66.7%). The average number of organs harvested per donor was 2.8, and this ratio was smaller for donors with expanded criteria compared to other donors.

**Conclusion:**

In most cases, potential organ donors died of brain death, were older than middle age, were male and were victims of a hemorrhagic stroke. The majority of the donors were expanded criteria donors and donated an average of two to three organs. The organs donated most frequently were the kidneys and liver.

## INTRODUCTION

Organ failure is associated with high rates of morbidity and mortality and high health care costs.^(^^1^^)^ The shortage of organs for transplantation is a serious medical and social problem because transplantation is often the only therapeutic option for organ failure.^(^^2^^)^ Because of the difference in the supply and demand of donated organs, transplant waiting lists continue to grow worldwide.^(^^1^^)^

In 2014, Portugal ranked fourth among the European Union member states in the number of organ donations, with 27 donors per million inhabitants.^(^^2^^)^

For centuries, death was defined as irreversible cardiac arrest.^(^^3^^)^ The development of organ support systems in recent decades led to a need to define a new concept: brain death (BD).^(^^3^^)^ BD is the cessation of secondary brain functions due to a known and irreversible cause.^(^^4^^)^

Deceased organ donors are the main source of solid organs for transplantation.^(^^1^^)^ Victims with BD or a stopped heart are considered eligible donors.^(^^5^^)^

In recent years, advancements in organ preservation techniques have allowed for an increase in the number of donors and have ensured the proper functioning of organs until their harvest and transplantation.^(^^6^^)^ In this respect, many countries recognize that organ donation is one of the components of end-of-life care for all patients who die in an intensive care unit (ICU).^(^^2^^)^

Previously, the ideal organ donor was a young patient with a traumatic brain injury (TBI). However, because of advancements in health care and the resulting increase in the average life expectancy, donors are increasingly older, and most are victims of sudden illnesses. At present, age is not an exclusion criterion for donation, and there is an increasing trend in the number of people classified as expanded criteria donors (ECDs), that is, older individuals with comorbidities who are also considered potential organ donors (PDs).

These changes in age and etiological factors indicate a need to identify the profile of organ donors, as early detection may be the most effective approach to preventing the loss of donation opportunities. The objectives of this study are to clinically and demographically characterize PDs admitted to a general ICU, identify reasons for non-donation and determine the numbers and types of donated organs.

## METHODS

This descriptive and retrospective study included patients admitted to a general ICU as PDs from January 2010 to December 2015.

The data were collected by analyzing medical records. The following demographic and clinical variables were analyzed: number of admissions, distribution of PDs and effective donors (EDs) by gender and age, number of non-effective donors (NEDs), causes of non-donation, causes of BD, blood types, comorbidities, patient origins, number of donated organs and tissues, number of organs per donor and duration of each donation phase.

Brain death was determined according to the brainstem death criterion adopted in Portuguese legislation, which includes the following items: (1) previous clinical status (deep coma, absence of spontaneous breathing, knowledge of the cause and irreversibility of the clinical condition, exclusion of conditions that could be responsible for the suppression of the functions referred to in the previous assumptions); and (2) diagnostic criteria (deep coma on the three-point Glasgow coma scale, absence of brain stem-dependent reflexes [photomotor, corneal, oculocephalic, oculovestibular and pharyngeal] and absence of spontaneous breathing using the apnea test).^(^^3^^)^

Brain death was confirmed by using at least two sets of tests, with an interval between them that was appropriate to the clinical condition and age, with 2 h set as the minimum expected interval between each set of tests for adult patients.^(^^3^^)^ In December 2017, the Federal Medical Council of Brazil published new criteria for BD that required a minimum interval of 1 h between each set of tests in adults;^(^^7^^)^ however, the older criteria were used in this study.

Potential organ donors were victims of neurological catastrophes who had confirmation of BD. They were considered EDs after donating organs or tissues or NEDs if they presented with donor-exclusion factors or unfavorable intraoperative conditions.

Expanded criteria donors were 50 years of age or older and presented with two of the following three criteria: arterial hypertension, death of a vascular cause or serum creatinine >1.5mg/dL.

Statistical analyses were conducted using Statistical Package for Social Science (SPSS) version 24.0 software. The following tests were used: Student's *t*-test (parametric) or the Mann-Whitney test (non-parametric) for comparing two independent samples (age group vs. gender or type of donor [ECDs and others]); the chi-square test to correlate nominal variables (gender and cause of BD); the Kruskal-Wallis test (non-parametric) to test differences between three or more groups in independent samples (age and cause of BD, time and cause of BD); Spearman's rho correlation coefficient for comparing scalar variables after confirming non-normal distributions (number of harvested organs and age, duration of collection and number of harvested organs); and the Pearson correlation coefficient for comparing numerical variables (number of harvested organs and age for each type of donor [ECDs and others]). P-values < 0.05 (95% confidence interval) were considered statistically significant.

## RESULTS

**Figure 1:**
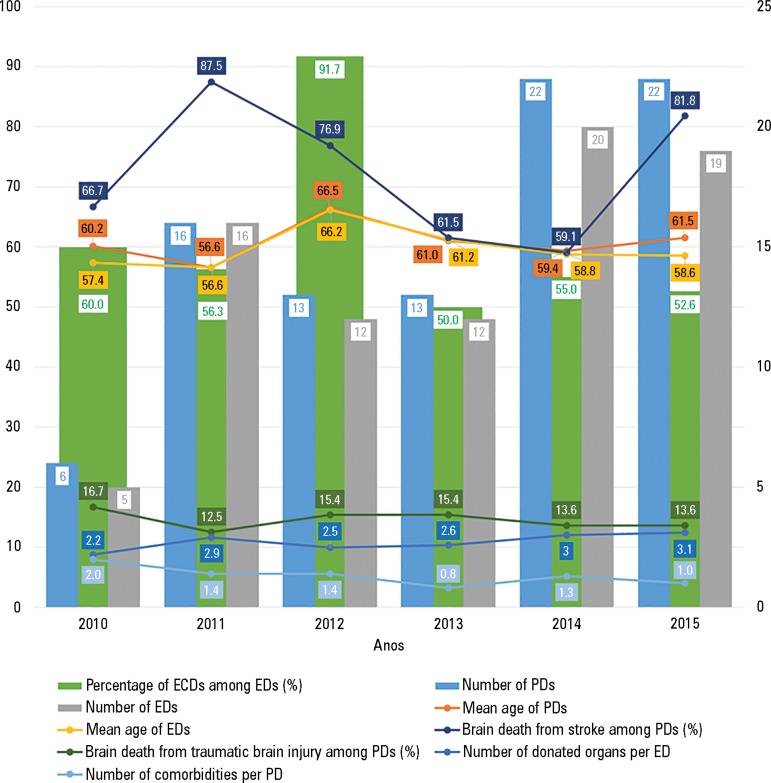
Distribution of the number of potential and effective donors from 2010-2015 and their profiles. ECD - expanded criteria donor; ED - effective donor; PD - potential donor.

A total of 92 PDs were admitted from 2010 to 2015, and the highest number of cases was recorded in the last two years of the study (Figure 1). There were eight NEDs (8.7%) and 84 EDs (91.3%). The reasons for non-donation among the NEDs were registration in the National Register of Non-Donors (Registo Nacional de Não Doadores-RENNDA) (one case), infection with human immunodeficiency virus (one case), diagnosis of herpetic encephalitis (one case), intraoperative refusal (five cases, including macroscopic evidence of tumor [two cases] and macroscopic aspects and poor perfusion of organs [three cases]). The results for PDs and EDs will be described in detail below.

### Potential donors

The mean age of the PDs was 60.7 ± 13.5 years, and this mean was higher in women (64.0 ± 14.3 years) than in men (57.8 ± 12.3 years) (p = 0.071). The mean age varied over the years with no linear trend.

Forty-three donors were women (46.7%), and 49 were men (53.7%).

In all cases, the diagnosis of BD was confirmed by clinical tests.

The most common causes of BD were hemorrhagic stroke (55.4%), ischemic stroke (17.4%), TBI (14.1%), anoxic brain injury after resuscitation from cardiac arrest (7.6%), spontaneous acute subdural hematoma (2.2%), spontaneous subarachnoid hemorrhage (2.2%) and hydrocephalus secondary to a Chiari type 1 malformation (1.1%). The leading causes of TBI were falls (61.5%), car accidents (23.1%) and firearm accidents (15.4%). In all NEDs with intraoperative refusal, the cause of BD was a cerebrovascular etiology (ischemic or hemorrhagic stroke). With respect to the two major causes of BD (stroke and TBI), despite the variability in the percentages over the years, vascular causes were more prevalent in all cases.

The mean age was lower in PDs with TBI (45.8 ± 12.2 years), hemorrhagic stroke (64.3 ± 11.2 years) and ischemic stroke (67.7 ± 8.9 years). The differences in the age distribution of PDs according to the cause of BD were significant (p = 0.000).

With respect to the distribution of the major causes of BD by gender, most TBI cases occurred in men (76.9%). Ischemic stroke was slightly more common in women (56.2%) than in men (46.8%), whereas hemorrhagic stroke was slightly more common in men (54.9%) than in women (45.1%). However, these differences were not significant (p = 0.191).

The distribution of ABO and Rh blood types was evaluated in PDs. The most common types were A Rh+ (43.5%), O Rh+ (30.4%), O Rh- (13.0%), A Rh- (8.7%), B Rh+ (3.3%) and AB Rh+ (1.1%). None of the evaluated cases had blood types B Rh- or AB Rh-.

The most common comorbidities were hypertension (43.3%), atrial fibrillation (AF) (34.8%), alcoholism (18.5%), diabetes mellitus (15.2%), dyslipidemia (13.0%), heart and/or cerebrovascular disease (6.5%) and smoking (6.5%). The mean number of comorbidities ranged from 0.8 to 2.0 for each PD. Approximately one-third (34.8%) of the PDs had at least two comorbidities, and 13.0% had at least three comorbidities. Furthermore, 69.2% of the PDs with TBI had no comorbidities.

With regard to patient origin, more than half (60.9%) came from the emergency department and the remainder came from the stroke unit (21.7%), an infirmary (7.6%), surgery unit (6.5%) or coronary ICU (3.3%).

The mean time from ICU admission to diagnosis of BD was 32 h and 8 min (± 29 h and 28 min). For the three major causes of BD, the mean time was 29 h and 10 min (± 29 h and 28 min) for TBI, 30 h and 7 min (± 24 h and 18 min) for hemorrhagic stroke and 31 h and 16 min (± 28 h and 42 min) for ischemic stroke. There were no significant differences (p = 0.948) between these times and the causes of BD.

### Effective donors

The mean age of EDs was lower than that of PDs (59.7 ± 13.3 years), and the mean age was higher in women (62.3 ± 14.0 years) than in men (57.4 ± 12.4 years) (p = 0.089). In the last three years of the study, there was a decreasing trend in the mean age of EDs.

The gender distribution for EDs was similar to that of PDs: 39 (46.4%) were women and 45 (53.6%) were men.

Of the 84 EDs, the majority (59.5%) were ECDs: 43 (51.2%) were aged >60 years, and 7 (8.3%) were aged 50-59 years and presented with two of the evaluated criteria (presence of arterial hypertension and stroke as the cause of BD). The mean age was higher in ECDs (68.6 ± 7.7 years) than in other EDs (46.5 ± 7.7 years, p = 0.000). Among ECDs, the mean age was higher in women (70.6 ± 7.9 years) than in men (66.5 ± 7.0 years, p = 0.057).

The organs most frequently harvested were the kidneys (84.5%), liver (66.7%), heart (22.6%), lungs (9.5%) and pancreas (7.1%) (Table 1). Only tissues were collected from three EDs. In the analyzed period, 236 organs were harvested, giving a ratio of 2.8 organs per donor (Table 2). The number of organs per donor per year varied from 2.2 to 3.1, and higher rates were not always associated with donors having a lower mean age. In ECDs, the number of harvested organs was smaller than in other EDs (2.3 ± 1.0 versus 3.5 ± 1.7 organs per donor, respectively; p = 0.001).

**Table 1:** Organs harvested and their respective distribution by effective donors.

Effective organ donors	Harvested organs				
	Kidneys	Liver	Heart	Lungs	Pancreas
31	√	√	-	-	-
18	√	-	-	-	-
9	-	√	-	-	-
7	√	√	√	-	-
5	√	-	√	-	-
4	√	√	√	√	√
2	√	√	-	√	-
3	-	-	-	-	-
1	-	-	√	-	-
1	√	√	-	-	√
1	√	√	√	-	√
1	√	√	√	√	-
1	√	-	-	√	-
84 N (%)	71 (84.5)	56 (66.7)	19 (22.6)	8 (9.5)	6 (7.1)
236 organs	140	56	19	15	6

**Table 2:** Comparison between national data and hospital data collected from 2010 to 2015 on effective donors.

Year	Centro Hospitalar Tondela-Viseu				National data (National Transplantation Coordination)^(^^9^^)^			
	Effective donors n (%)	Mean age (years)	Harvested organs n (%)	Number of organs per donor	Effective donors n	Mean age (years)	Harvested organs n	Number of organs per donor
2015	19 (6.0)*	58.6	59 (6.6)†	3.1	318	54.2	894	2.8
2014	20 (6.9)*	58.8	59 (7.1)†	3.0	289	51.4	0.83	2.9
2013	12 (4.1)*	61.2	31 (3.6)†	2.6	295	53.7	859	2.9
2012	12 (4.8)*	66.2	30 (4.0)†	2.5	252	53.3	749	3.0
2011	16 (5.3)*	56.6	46 (5.0)†	2.9	301	48.7	928	3.1
2010	5 (1.5)*	57.4	11 (1.2)†	2.2	323	51.3	0.83	2.9
Total	84 (4.7)*	59.7	236 (4.5)†	2.8	1,778	52.1	5,187	2.9

* Annual percentage of effective donors compared with national data;

† annual percentage of harvested organs compared with national data.

Only tissues were harvested from 68 EDs (81.0%). The tissues most commonly harvested were the corneas (77.9%), blood vessels (55.9%) and heart valves (2.9%).

The time interval from the diagnosis of BD to organ harvesting was 5 h and 39 min (± 3 h and 18 min), and the mean total length of stay in the ICU was 37 h and 47 min (± 29 h and 4 min).

There was a significant inverse correlation between the number of harvested organs and the age of EDs, i.e., the older the age, the smaller the number of harvested organs (Rho = -0.450; p = 0.000). Similarly, there was a significant inverse correlation between the number of harvested organs and the age of ECDs (p = 0.000) and other EDs (p = 0.028).

There was a non-significant positive association between the length of stay in the ICU and the number of harvested organs, i.e., the longer the stay, the larger the number of harvested organs (Rho = 0.091; p = 0.408).

## DISCUSSION

A predominance of male PDs with a lower mean age was observed. The primary etiology of BD was cerebrovascular disease, which was associated with a higher mean age. Common comorbidities included arterial hypertension and AF, which are both risk factors for cerebrovascular diseases. Most EDs were ECDs, and the number of organs per donor was smaller in the latter group. The most commonly harvested organs were the kidneys and liver, and the number of harvested organs varied inversely with age.

One study published in 2013 identified a change in the profile of EDs and found that the leading cause of BD was stroke (55%) and that the second main cause was TBI (35%).^(^^8^^)^

However, few studies to date have described PDs or EDs, and therefore, the comparison of our sample with that of other series is limited. Nevertheless, we deemed it pertinent to compare our results with national data from the Portuguese Institute of Blood and Transplantation (Instituto Português do Sangue e da Transplantação-IPST).^(^^9^^)^

The number of EDs in our sample increased steadily in recent years, and the group's representation in national data has increased. The comparison between the first and last years of the analyzed period indicated that the mean age increased in our sample and at the national level, although this increase was not linear over the years.

Most PDs and EDs were men, and they had a lower mean age than women. This result may be explained by the higher cardiovascular risk in men in younger age groups. Another contributing factor is that the average life expectancy is higher in women. The mean age was lower in PDs with TBI, and these results were expected because TBIs are sudden and unexpected events, in contrast with clinical causes, which usually occur in patients with comorbidities and at older ages.

As observed at the national level,^(^^9^^)^ the causes of most cases of BD in our sample were clinical, while traumatic etiologies were less common. However, traumatic causes may be underrepresented in our sample because, for several years, TBI victims were referred to other centers due to a lack of availability of neurosurgery teams at the center studied here.

The leading cause of BD in our sample was stroke, and stroke is a major cause of death in the general population in Portugal.

Most PDs had blood types A (52.2%) and O (43.4%), which is consistent with the distribution of blood types in the Portuguese population.^(^^10^^)^

Considering that the main cause of BD was cerebrovascular disease, it was expected that all of the identified comorbidities would be risk factors for its occurrence. Moreover, the number of patients with a history of alcoholism was high in our sample, probably because the district of Viseu is located in a wine region (Dão Lafões and Douro).

The most common place of origin was expected to be the emergency department, which is the main entry point to medical care for patients with trauma or sudden illness.

In our sample, the rate of utilization of PDs was high because Portuguese law is based on presumed consent for organ donation. As such, all national citizens, stateless persons and foreigners residing in Portugal who have not registered their status as non-donors to the Ministry of Health are potential postmortem donors.^(^^11^^)^

The higher number of kidneys and livers harvested compared to other organs is consistent with national data^(^^9^^)^ and may be explained by the percentage of ECDs in our sample.

The evaluation of the length of stay of the PDs in the ICU allows for reflection on the use of material and human resources. Because this ICU belongs to a hospital intended for organ donors but not organ recipients, it is important to note that the time from the diagnosis of BD to organ harvesting depends on the availability of collection teams and the time required for transport.

This study has several limitations: its retrospective nature does not allow for the exclusion of bias related to the referral of patients with neurological catastrophes to the ICU team or the Hospital Donor Coordination team, considering that referrals depend on each professional and are based on individual decisions. In addition, the small sample size limited us from drawing further conclusions and prevented the detection of significant differences in infrequent results. However, the fact that the results agree with the national data suggests that our sample is representative. In addition, it was not possible to determine how many harvested organs were transplanted and the rate of graft survival, and these data may be highly relevant.

## CONCLUSION

At present, potential organ donors are most often male donors who died of brain death following admission to the emergency department for hemorrhagic stroke. Their mean age was 56.6 to 66.2 years, the most common blood type was A Rh+, and they generally had a history of arterial hypertension. It was found that potential organ donors can become effective donors with expanded criteria. The kidneys and liver were harvested in most cases, and each donor donated an average of two to three organs.
